# Automatic clustering method to segment COVID-19 CT images

**DOI:** 10.1371/journal.pone.0244416

**Published:** 2021-01-08

**Authors:** Mohamed Abd Elaziz, Mohammed A. A. Al-qaness, Esraa Osama Abo Zaid, Songfeng Lu, Rehab Ali Ibrahim, Ahmed A. Ewees

**Affiliations:** 1 Hubei Engineering Research Center on Big Data Security, School of Cyber Science and Engineering, Huazhong University of Science and Technology, Wuhan, China; 2 Department of Mathematics, Faculty of Science, Zagazig University, Zagazig, Egypt; 3 State Key Laboratory for Information Engineering in Surveying, Mapping and Remote Sensing, Wuhan University, Wuhan, China; 4 Department of Mathematics, Faculty of Science, Seuz University, Suez, Egypt; 5 Department of Computer, Damietta University, Damietta, Egypt; Hefei University of Technology, CHINA

## Abstract

Coronavirus pandemic (COVID-19) has infected more than ten million persons worldwide. Therefore, researchers are trying to address various aspects that may help in diagnosis this pneumonia. Image segmentation is a necessary pr-processing step that implemented in image analysis and classification applications. Therefore, in this study, our goal is to present an efficient image segmentation method for COVID-19 Computed Tomography (CT) images. The proposed image segmentation method depends on improving the density peaks clustering (DPC) using generalized extreme value (GEV) distribution. The DPC is faster than other clustering methods, and it provides more stable results. However, it is difficult to determine the optimal number of clustering centers automatically without visualization. So, GEV is used to determine the suitable threshold value to find the optimal number of clustering centers that lead to improving the segmentation process. The proposed model is applied for a set of twelve COVID-19 CT images. Also, it was compared with traditional k-means and DPC algorithms, and it has better performance using several measures, such as PSNR, SSIM, and Entropy.

## Introduction

Coronavirus (COVID-19) that first reported in December 2019, in Wuhan, China, has been spread to more than 200 countries and regions. It could be transmitted through the respiratory droplets and the contact [1, 2]. Diagnosing COVID-19 is a critical challenge for health organizations that must be accurately and efficiently implemented to make necessary plans [3]. The real-time polymerase chain reaction (RT-PCR) can be used to diagnose COVID-19, but it is a time-consuming test; also, it may suffer from false-negative diagnosing [4, 5]. Therefore, medical imaging, such as chest X-ray and chest Computed Tomography (CT) can be used efficiently for diagnosing COVID-19.

Image segmentation is considered as an important key for analyzing medical images. Its main goal is to distinguish the region of interest (ROI) from the area of outside. Moreover, it also enables to extract important features, for example, texture, and shape of tissues [6–8]. Recent advances in the field of medical imaging show that medical images can be heavily used in many medical procedures. Therefore, huge numbers of medical images are generated every day. With this massive volume of images, it is a big challenge for analyzing and diagnosing since manual segmentation requires more time; more so, it may not meet the demand of analyzing big images data.

To this end, creating automatic methods for medical image segmentation is an important and urgent issue. Therefore, in recent decades, many efforts have been made by researchers to propose various medical image segmentation methods using various technologies, for example, region-based methods, clustering methods, threshold algorithms, machine learning, deep learning techniques, and others. The segmentation of Computed Tomography (CT) images is a critical step in Computer-Aided Diagnosis (CADx) systems. Therefore, many studies have been proposed, such as Dev et al. [9] proposed a lung cancer detection from DICOM CT images using the support vector machine (SVM) algorithm. The tested images could be classified as cancerous or non-cancerous. Shakeel et al. [10] applied a profuse clustering technique (PCT) to segment lung CT images and then employed a deep learning model to detect lung cancers from the tested CT images. Medeiros et al. [11] presented a segmentation method based on active contour method (ACM) with fuzzy border detector to segment lung CT images. Wang et al. [12] presented CT image segmentation method based on adaptive fully dense(AFD) neural network. Their proposed method had been evaluated using CT images of liver cancer. More so, they showed that this method could successfully segment CT images with complex boundaries. Sousa et al. [13] proposed an automatic CT images segmentation method for lung and trachea. Their proposed method, called ALTIS showed good performance in detecting abnormal structures in CT images. Ye et al. [14] proposed a heart CT image segmentation method using multi-depth fusion network. Sun et al. [15] proposed convolutional neural networks (CNN) model to classify CT images, moreover, to segment eyes, and the surrounding organs. Li et al. [16] utilized the power of blockchain technology for medical image segmentation. Paulraj et al. [17] proposed a possibilistic fuzzy C‐means method for lung CT images segmentation. Han et al. [18] used generative adversarial networks (GANs) for object detection in lung nodules.

Chen et al. [19] proposed a dictionary-based method for automatically segment 3D CT images of pathological lungs. Shariaty et al. [20] used a thresholding algorithm to segment lung CT images. Day et al. [21] proposed a lung segmentation approach to identify lung diseases using CT images. They used an enhanced graph cuts algorithm and Gaussian mixture model (GMM). Swierczynski et al. [22] proposed a mathematical model for lung CT image segmentation. The proposed level-set formulation combines active dense displacement estimation with Chan–Vese segmentation. Sousa et al. [13] presented a segmentation method called ALTIS to segment lung and trachea in CT images.

Among all the mentioned methods, deep learning approaches have received wide popularity because of their notable performance in image segmentation. However, these methods require extensive training using many images [23], and this may cause a problem for some applications that have only limited images. Therefore, unsupervised methods, such as clustering, are preferable since they do not require more images for training. There are several types of clustering segmentation methods used for medical images, for example, fuzzy C-means [24], density-based clustering [25], and K-means [26].

According to Tao et al. [27] Chest CT is more sensitive to diagnose COVID-19 comparing to RT-PCR (initial reverse-transcription polymerase chain reaction). Therefore, in this paper, we propose a clustering method to segment chest CT images of infected people of COVID-19.

In this study, we apply density peaks clustering (DPC) [28, 29] based on generalized extreme value distribution to deal with chest CT scans of COVID-19. Based on visual selection rule of density peaks clustering and following [30], the clustering point has a higher density than other points with a relative large distance between each of them. Moreover, the measure that used to determine the clustering center is approximately the generalized extreme value (GEV) distribution [31]. Whereas, the upper quantile of GEV is used to detect the clustering is higher. The main motivation to combine the DPC and GEV is to benfit from the strength of DPC that avoids the limitations of iteration clustering methods. In addition, using GEV to determine the optimal number of clustering in automatic form.

The contributions of this study are as follows:

- Present an image segmentation model to segment COVID-19 CT images using a density peaks clustering based on generalized extreme value distribution.
- The proposed model was evaluated with a set of twelve CT images of COVID-19 collecting form different datasets.
- To evaluate our model, we compared it with density peaks clustering and k-means clustering methods, and it showed better performances.

## Materials and methods

### Density peaks clustering

In this section, the basic concepts of clustering by finding the peaks of density (DPC) algorithm is introduced [28]. In general, the main hypothesis in DPC is assumed that centers of clusters have a higher density than their neighbors, as well as, the distance between those centers is large.

Considering the dataset is given by *X* = [*x*_1_, *x*_2_, …, *x*_*n*_] has *n* samples. The local density *ρ*_*i*_ of *x*_*i*_ can be computed as:

$$\begin{array}{c} \rho_{i}=\sum_{i=1}^{N}\xi (d_{ij}-d_{c}), \end{array}$$ (1)

where *d*_*ij*_ is the distance between *x*_*i*_ and *x*_*j*_, while *d*_*c*_ refers to the cut-off distance. *ξ* represents the kernel function and it is defined as:

$$\begin{array}{c} \xi =\{\begin{array}{c} 1,\;x_{i}>0\\ 0,\;otherwise \end{array}, \end{array}$$ (2)

Moreover, the minimum distance between *x*_*i*_ and other points of higher *ρ* is represented by *δ*_*i*_ and it is defined as:

$$\begin{array}{c} \delta_{i}=\{\begin{array}{c} min_{j:\rho_{j}>\rho_{i}}\left(d_{ij}\right),\;\;\exists \rho_{j}>\rho_{i}\\ max_{j}\left(d_{ij}\right),\;otherwise \end{array}, \end{array}$$ (3)

The points that have large *δ* and high *ρ* are considered as clustering centers. However, each of the rest points is assigned to the nearest center. According to these behaviors, the DPC algorithm is faster than other clustering methods that need more iterations to find the optimal cluster centers.

In some cases, the class may have two high-density points with a small distance between each them, and to avoid splitting the class into small sub-classes, there is another measure that is used which consider both *ρ* and *δ* together and it is defined as:

$$\begin{array}{c} \theta =\min(\rho^{*},\delta^{*}) \end{array}$$ (4)

where ρ* and δ* refer to the normalization of ρ* and δ*, respectively and they are formulated as:

$$\begin{array}{c} \rho^{*}=\frac{\rho -\rho_{min}}{\rho_{max}-\rho_{min}},\;\delta^{*}=\frac{\delta -\delta min}{\delta max-\delta min} \end{array}$$ (5)

The clustering centers have *θ* higher than other points.

### Generalized extreme value distribution

This section presented the mathematical notation of the generalized extreme value (GEV) distribution [31]. In general, the GEV is considered as a generalized family of the Gumbel, Fréchet and Weibull using single parameter and is defined as:

$$\begin{array}{c} H\left(x;k,\sigma ,\mu \right)=\exp\{-{\left(1+k\left(\frac{x-\mu }{\sigma }\right)\right)}^{\frac{-1}{k}}\},\left(1+k\left(\frac{x-\mu }{\sigma }\right)\right)>0 \end{array}$$ (6)

where *μ*, *σ* and *k* represent the location, scale and shape parameter, respectively. The maximum likelihood estimation is used to estimate these parameters which defined as:

$$\begin{array}{c} \ell \left(x;\mu ,\sigma ,k\right)=-n\log\sigma -\left(1+\frac{1}{k}\right)\sum_{i=1}^{n}\log\left(1+k\left(\frac{x_{i}-\mu }{\sigma }\right)\right)\\ -\sum_{i=1}^{n}{\left(1+k\left(\frac{x_{i}-\mu }{\sigma }\right)\right)}^{-\frac{1}{k}} \end{array}$$ (7)

For determining the MLEs of the parameters (*μ*, *σ*, *k*) we can for any given data set the maximization is straightforward using standard numerical optimization algorithms for solving the following equations:

$$\begin{array}{c} \frac{\partial \ell }{\partial \mu }=\left(k+1\right)\sum_{i=1}^{n}\frac{1}{1+A_{i}}-\sum_{i=1}^{n}\frac{1}{{\left(1+A_{i}\right)}^{1+\frac{1}{k}}}=0 \end{array}$$ (8)

$$\begin{array}{c} \frac{\partial \ell }{\partial \sigma }=\left(k+1\right)\sum_{i=1}^{n}\frac{A_{i}}{1+A_{i}}-\sum_{i=1}^{n}\frac{A_{i}}{{\left(1+A_{i}\right)}^{1+\frac{1}{k}}}-nk=0 \end{array}$$ (9)

$$\begin{array}{c} \frac{\partial \ell }{\partial k}=\sum_{i=1}^{n}\log\left(1+A_{i}\right)+\sum_{i=1}^{n}{\left(1+A_{i}\right)}^{\frac{-1}{k}}\sum_{i=1}^{n}\frac{A_{i}}{1+A_{i}}-\left(k+1\right)\sum_{i=1}^{n}\frac{A_{i}}{1+A_{i}}\\ -\sum_{i=1}^{n}\frac{1}{{\left(1+A_{i}\right)}^{\frac{1}{k}}}\sum_{i=1}^{n}\log\left(1+A_{i}\right)=0\;\; \end{array}$$ (10)

Where $A_{i}=k\left(\frac{x_{i}-\mu }{\sigma }\right)$ in (7).

Thereafter, using this estimation to obtained the quantile ${\hat{x}}_{p}$ can be defined as:

$$\begin{array}{c} {\hat{x}}_{p}=\{\begin{array}{c} \hat{\mu }-\frac{\hat{\sigma }}{\hat{k}}(1-y_{p}^{-\hat{k}}),\;\hat{k}\neq 0\\ \hat{\mu }-\hat{\sigma }\log\left(y_{p}\right),\;\hat{k}=0 \end{array},y_{p}=-\log\left(p\right) \end{array}$$ (11)

where *p* represents the probability of quantile. Therefore, the *x*_*i*_ is considered as a clustering center when the following condition is satisfied.

$$\begin{array}{c} \theta_{i}>{\hat{x}}_{p} \end{array}$$ (12)

### Proposed COVID-19 image segmentation model

**Fig 1:**
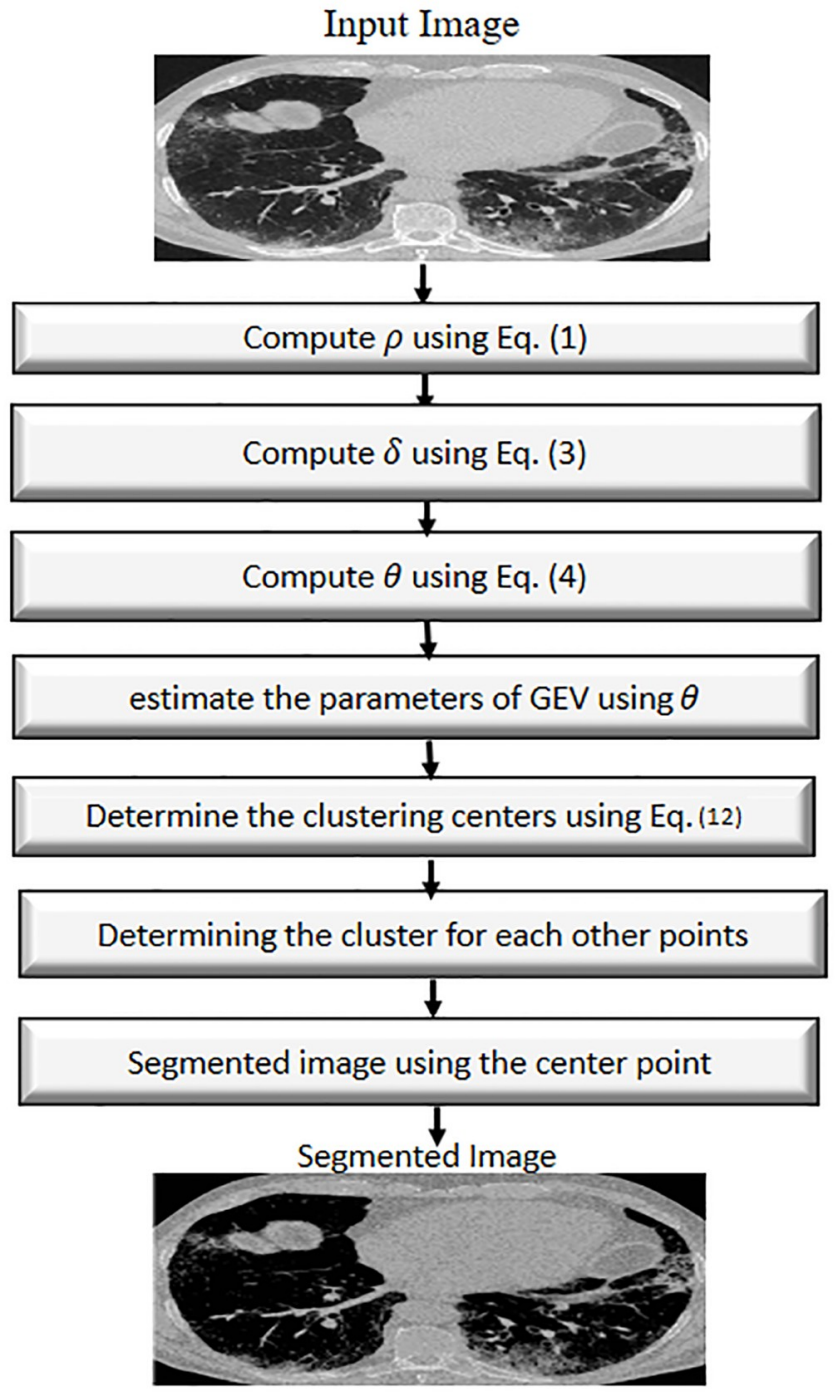
Steps of proposed COVID-19 image segmentation method.

In this section, the proposed model that used to tackle the problem of segmented the COVID-19 image using the density peak clustering based on generalized extreme value is introduced. The proposed model starts by reading the image and computing the value of *ρ* and *δ* using Eqs (1) and (3), respectively. Thereafter compute the value of *θ* using Eq (4) and using the maximum likelihood method to estimate the parameters of GEV using *θ* as input for it. Followed by applying Eq (12) to determine the clustering centers and determining the cluster for each other points. In the case, the distance between cluster center and current point is less than *δ*_*i*_ then assigned the current point to the cluster center. The steps of the proposed model are given in Fig 1.

## Experiment and results

### Dataset

**Fig 2:**
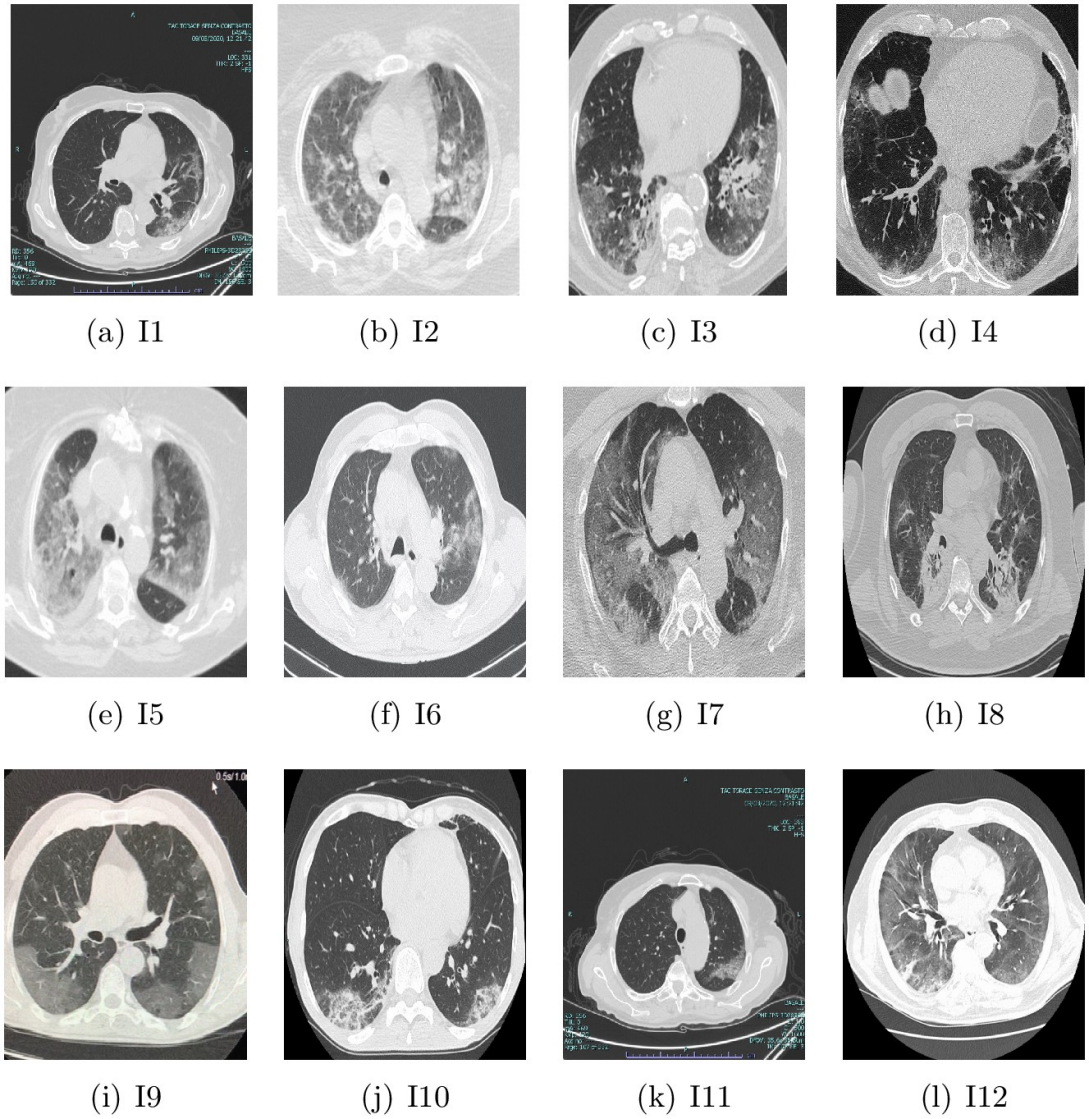
Original COVID-19 images.

To assess the quality of the segmentation method for COVID-19 CT images, a set of twelve image is used from [32]. These images are collected from different datasets such as CheX aka CheXpert [33], OpenI [34], Google [35], PC aka PadChest [36], NIH aka Chest X-ray14 [37], and MIMIC-CXR [38]. The images are resized to 224x224 pixels [32]. Fig 2 depicts the sample of the tested image which contains twelve COVID-19 images.

### Performance measure of segmentation

Three measures are used to assess the performance of all algorithms to evaluate the quality of the segmentation process. These measures are peak signal-to-noise ratio (PSNR) [39] as in Eq (13), the structural similarity index (SSIM) as in Eq (14) [40], and entropy as in Eq 15.

$$\begin{array}{c} PSNR=20\log_{10}\left(\frac{255}{RMSE}\right),\;\;\\ RMSE=\sqrt{\frac{\sum_{i=1}^{M}\sum_{j=1}^{Q}{\left(I\left(i,j\right)-I_{s}\left(i,j\right)\right)}^{2}}{M\times Q}} \end{array}$$ (13)

where *I* and *I*_*s*_ determine the image and its segmented version, respectively at the size *M* × *Q*.

$$\begin{array}{c} SSIM\left(I,I_{s}\right)=\frac{\left(2\mu_{I}\mu_{I_{s}}+c_{1}\right)\left(2\sigma_{I,I_{s}}+c_{2}\right)}{\left(\mu_{I}^{2}+\mu_{I_{s}}^{2}+c_{1}\right)\left(\sigma_{I}^{1}+\sigma_{I_{s}}^{2}+c_{2}\right)} \end{array}$$ (14)

where *μ*_*I*_ and $\mu_{I_{s}}$ determine the average intensity of the *I* and *I*_*s*_, respectively. *σ*_*I*_ and $\sigma_{I_{s}}$ determine the standard deviation values for the *I* and *I*_*s*_, respectively. Covariance of *I* and *I*_*s*_ is presented by $\sigma_{I,I_{s}}$. *c*_1_ is set to 6.5025 and *c*_2_ is set to 58.52252.

Moreover, the entropy of a discrete random variable is used to assess the quality of segmentation, and it is defined as:

$$\begin{array}{c} H\left(X\right)=-\sum_{x\in X}\;Prob\left(x\right)\;\log\left(Prob\left(x\right)\right) \end{array}$$ (15)

where *Prob* is a probability mass function.

### Results and discussion

**Fig 3:**
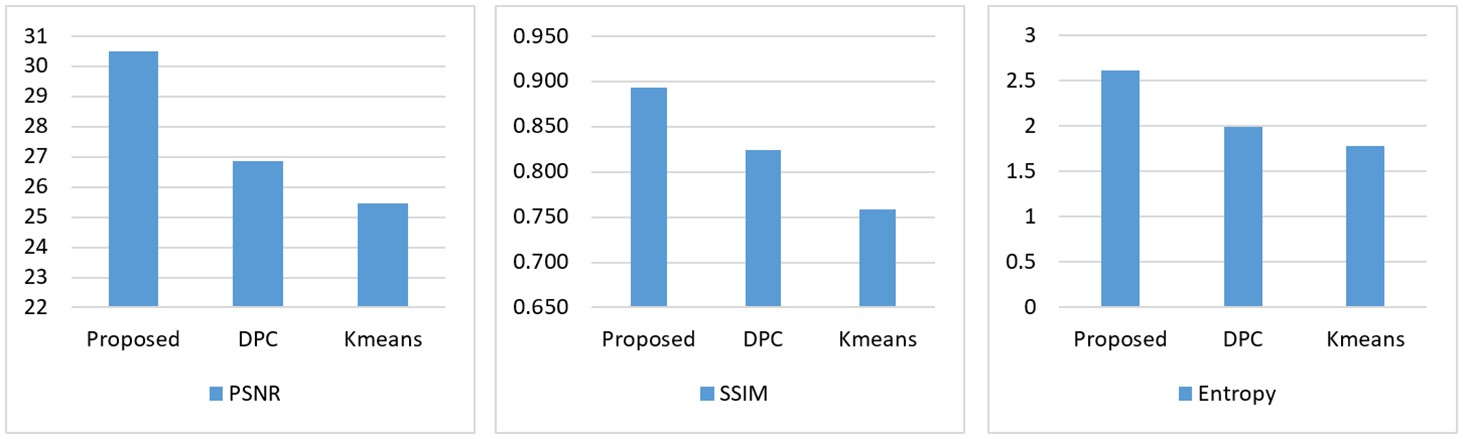
Average of the results of all measures.

This section shows the results of the proposed methods against the classical algorithm density peaks clustering (DPC) and K-means algorithm; these algorithms are widely used for processing medical images and clustering fields. The comparison uses three measures: PSNR, SSIM, and entropy for evaluating the algorithms using 12 images. Tables 1 and 2 and Fig 3 record these results. Table 1 depicts the number of clusters obtained by each method. To assess these obtained cluster centers we used PSNR, SSIM, and entropy.

**Table 1:** Number of clusters obtained by each algorithm.

Image	Proposed	DPC	K-means
Im1	5	3	6
Im2	11	9	10
Im3	7	6	5
Im4	6	4	9
Im5	6	7	5
Im6	7	2	5
Im7	5	3	5
Im8	6	3	2
Im9	4	2	3
Im10	4	2	3
Im11	4	5	6
Im12	5	4	3

**Table 2:** Segmentation results of all algorithms.

Name	Proposed			DPC			K-means		
	PSNR	SSIM	Entropy	PSNR	SSIM	Entropy	PSNR	SSIM	Entropy
Im1	39.2430	0.9908	4.1429	38.5551	0.9890	3.9253	26.8397	0.8332	1.7742
Im2	35.4548	0.9527	3.0468	24.2043	0.6870	0.7217	27.3187	0.7953	1.8065
im3	28.6461	0.9139	2.7085	26.9444	0.8532	1.9093	25.614	0.7542	1.7084
im4	32.6640	0.9425	2.9139	28.1261	0.8765	2.3435	23.7001	0.7247	1.8484
Im5	29.6723	0.9041	2.1994	28.5004	0.8915	2.4193	27.1021	0.8192	1.7120
Im6	32.0327	0.9457	2.5502	30.5735	0.9170	2.1647	26.7181	0.7309	1.8814
Im7	27.8433	0.8417	2.1388	27.7479	0.7906	1.8795	24.8568	0.6965	1.8639
Im8	25.1145	0.8161	2.3687	20.4955	0.6730	0.9318	25.595	0.7146	1.8095
Im9	25.1354	0.8451	1.8843	24.3401	0.8012	1.4831	26.0249	0.7536	1.8796
Im10	21.2405	0.7133	0.9849	18.9556	0.7143	1.5063	21.0752	0.683	1.7009
Im11	41.2158	0.9949	4.3850	32.1631	0.9478	3.1105	27.361	0.8666	1.6193
Im12	27.6072	0.8535	2.1303	21.5256	0.7508	1.5235	23.3786	0.7325	1.8377

From Table 2 that can be seen, the proposed method obtained the best PSNR results in 10 out of 12 images. In spite of the K-means obtained the best PSNR in two images, it is ranked last after DPC because it attained the better PSNR in 7 images in comparison with K-means, as shown in Fig 3.

In terms of the SSIM measure, the proposed method achieved the highest SSIM value in 11 out of 12 images, followed by DPC and K-means, respectively. That means, the proposed method can get the highest similarity with the original images than the other algorithms. As in Fig 3, the proposed method reached 89% of SSIM while the DPC and K-means reached 82% and 76%, respectively.

Regarding the entropy measure, the proposed method has higher image entropy than DPC and K-means algorithms. It outperformed them in 10 out of 12 images that lead to the best segmentation results. The rest of the algorithms are ranked as follows; the DPC reached the second rank while the K-means is ranked last.

**Fig 4:**
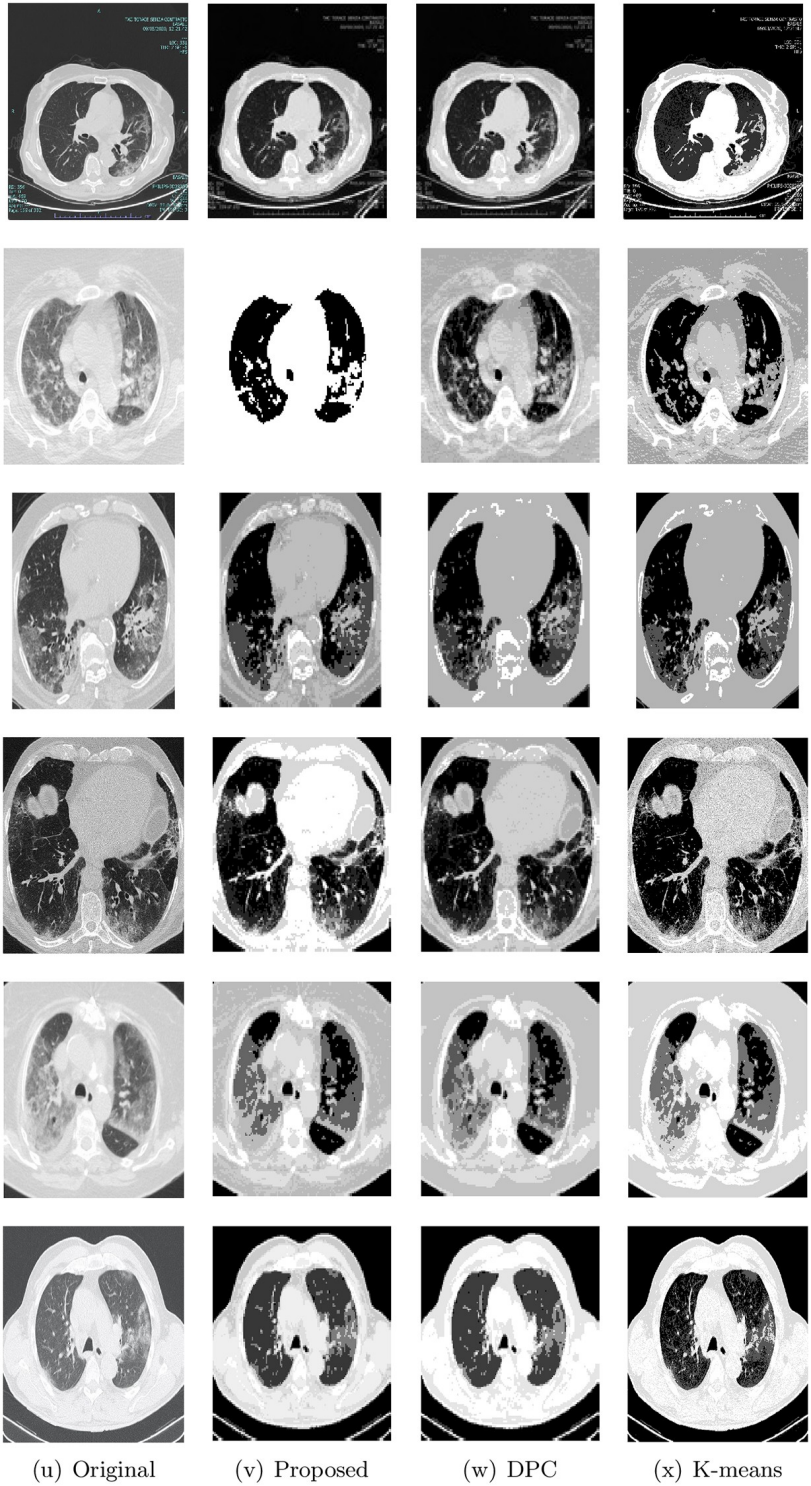
The segmented image of Im1 to Im6 based on the obtained results by (v)proposed method, (w) DPC, and (x) K-means.

**Fig 5:**
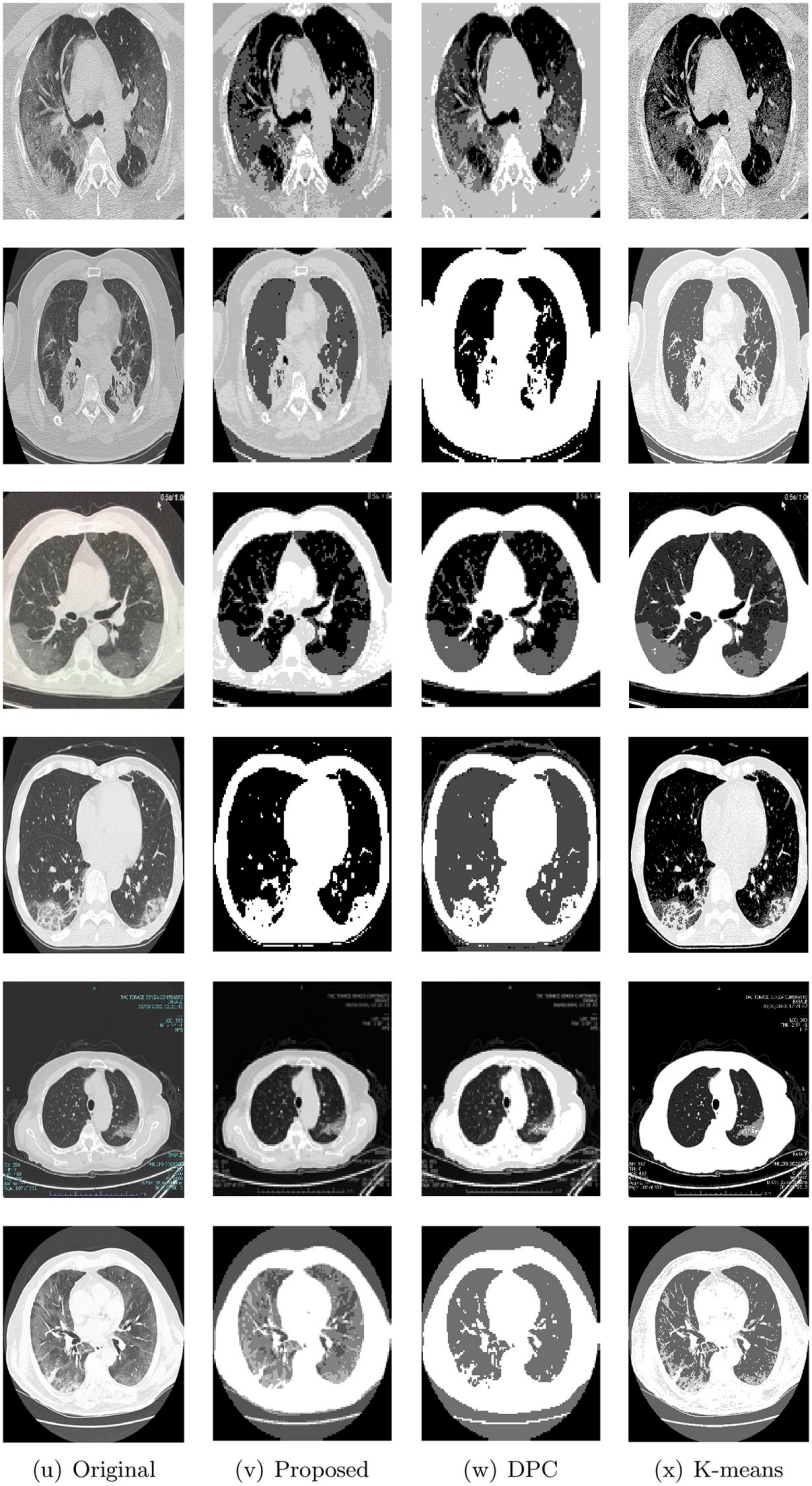
Original images Im7 to Im12 and their and the segmented results of the proposed method, DPC, and K-means.

Figs 4 and 5 shows the original images and the segmented results of the proposed method, DPC, and K-means. To display all images, the images are split into figures. From these figures, we can see that the proposed method produced better segmentation results in most of the images. These results indicate that the proposed method can efficiently segment the chest CT images with COVID-19.

From the previous analysis, it has been observed that the performance of the proposed model is better than the other two models. However, there are some limitations that affect its quality, such as processing time may be increased with increasing the size of a given image due to computing the pair-wise distance between the pixel of images.

## Conclusion

Analyzing medical images is very important for diagnosing diseases, and there are preliminary steps that needed to be implemented in image analysis process, such as image segmentation. The main work of segmentation methods in medical images is to find the region of interest (ROI) and to help in distinguishing it from outside regions. With the pandemic of COVID-19, it is necessary to find efficient segmentation methods that may help in improving the diagnosing process. Therefore, this paper proposes an efficient segmentation method for COVID-19 CT images. The proposed method uses density peaks clustering depending on generalized extreme value distribution. To test the performance of the proposed method, a set of twelve images of COVID-19 CT scans is used. The proposed method was compared to DPC and K-means clustering methods, and it showed better performances in terms of PSNR, SSIM, and entropy.
